# Inhibition of Caveolae Contributes to Propofol Preconditioning-Suppressed Microvesicles Release and Cell Injury by Hypoxia-Reoxygenation

**DOI:** 10.1155/2017/3542149

**Published:** 2017-09-19

**Authors:** Fan Deng, Shuang Wang, Shuyun Cai, Zhe Hu, Riping Xu, Jingjing Wang, Du Feng, Liangqing Zhang

**Affiliations:** ^1^Department of Anesthesiology, Affiliated Hospital of Guangdong Medical University, Zhanjiang, Guangdong, China; ^2^Department of Anesthesiology, Taihe Hospital, Hubei University of Medicine, Anesthesiology Research Institute of Hubei University of Medicine, Shiyan, Hubei Province, China; ^3^Department of Anesthesiology, Dongfeng General Hospital, Hubei University of Medicine, Shiyan, Hubei 442000, China; ^4^Key Laboratory of Protein Modification and Degradation, School of Basic Medical Sciences, Affiliated Cancer Hospital & Institute, Guangzhou Medical University, Guangzhou 511436, China; ^5^Guangdong Key Laboratory of Age-Related Cardiac and Cerebral Diseases, Institute of Neurology, Affiliated Hospital of Guangdong Medical University, Zhanjiang, Guangdong, China

## Abstract

Endothelial microvesicles (EMVs), released after endothelial cell (EC) apoptosis or activation, may carry many adverse signals and propagate injury by intercellular transmission. Caveolae are 50–100 nm cell surface plasma membrane invaginations involved in many pathophysiological processes. Recent evidence has indicated EMVs and caveolae may have functional effects in cells undergoing H/R injury. Propofol, a widely used anaesthetic, confers antioxidative stress capability in the same process. But the connection between EMVs, H/R, and caveolae remains largely unclear. Here, we found that H/R significantly increased the release of EMVs, the expression of CAV-1 (the structural protein responsible for maintaining the shape of caveolae), oxidative stress, and the mitochondrial damage, and all these changes were inhibited by propofol preconditioning. Interestingly, the caveolae inhibitor M*β*-CD strengthened the protective effect of propofol preconditioning. We further found that the release of EMVs is more significantly reduced under propofol preconditioning in the presence of the caveolae inhibitor M*β*-CD. EMVs released from H/R-treated cells caused a substantially increased mitochondrial and cellular damage to normal HUVECs after 4 hours of coculture. Thus, we conclude that inhibition of caveolae contributes to propofol preconditioning-suppressed microvesicles release and cell injury by H/R.

## 1. Introduction

The incidence of ischemia/reperfusion (I/R) injury associated with cardiovascular diseases increases gradually with age. Moreover, acute conditions like sepsis, trauma and shock, and various treatments such as organ transplantation and heart bypass surgery can lead to local or broad I/R injury [1]. During ischemia, loss of blood flow deprives tissue of oxygen and essential nutrients, and excessive oxygen free radicals generated following restoration of blood flow cause further (reperfusion) injury [2]. The improvement of excessive oxidative stress is a widely accepted therapeutic strategy for mitigation of I/R injury [3, 4].

Endothelial cells (ECs) are not only the target organs of I/R injury but also a major source of oxygen free radicals that damage the surrounding tissues [5]. Mitochondria are dynamic organelles that are exquisitely sensitive to damage from oxidative stress and are considered as both targets and producers of reactive oxygen species (ROS) [6]. It has been demonstrated that I/R-induced oxidative stress is a pivotal factor which leads to mitochondrial dysfunction [7]. I/R induced the upregulation of proapoptotic protein Bax and downregulation of antiapoptotic protein BCL2 which will lead to mitochondrial membrane permeability changes (the mitochondrial permeability transition pore (MPTP) opening, which in turn triggers apoptosis) and the loss of transmembrane potential [8]; then, cytochrome c and other proteins will be released from the mitochondria into the cytoplasm, which was considered to be a critical step in the mitochondrial apoptotic pathway [9]; finally, cytochrome c released into the cytoplasm induced caspase activation and cell apoptosis [10]. Therefore, maintaining EC and mitochondrial functional stability is a corking therapeutic strategy for mitigation of I/R injury.

Activation or apoptosis of ECs releases endothelial microvesicles (EMVs) between 100 nm and 1 *μ*m in diameter that express cell-specific surface antigenic epitopes and contain various proteins, mRNAs, miRNAs, and lipids [11]. These EMVs participate in the information exchange between cells [12]. Moreover, EMVs have been implicated in disease pathogenesis. For instance, EMVs may be involved in the pathogenesis of diabetes [13] and atherosclerosis [12] by conveying procoagulant, proinflammatory, and other pathogenic signals to surrounding cells and tissues [4, 14]. The number of EMVs released reflects the functional status of ECs and is positively correlated with apoptosis and EC damage. As oxidative stress, inflammation, and coagulation are critical for I/R injury, one possible therapeutic strategy is to inhibit EMV release from ECs during I/R.

Caveolae are 50–100 nm cell surface plasma membrane invaginations, which are rich in cholesterol and sphingolipids [15]. Caveolae consist of scaffolding proteins called caveolins. Caveolins are a 21 to 24 kDa family of membrane proteins that consist of three members; caveolin-1 (CAV-1) is abundantly expressed in endothelial cells, and caveolin-3 (CAV-3) is primarily expressed in muscle cells [16]. CAV-1, the structural protein responsible for maintaining the shape of caveolae, has been demonstrated that it acts as an important functional role in the modulation of several signal transduction pathways and processes during I/R [15, 17, 18]. CAV-1 can also be used as a marker protein for labeling the caveolae. However, whether the mitochondrial apoptotic pathway is involved in the specific mechanism of caveolae during I/R is not entirely clear.

Propofol is a popular general anesthetic, which has been extensively used in clinical anesthesia and sedation in critical patients because of its rapid onset and reversibility [19]. In myocardial cells, propofol preconditioning inhibits oxidative stress associated with the mitochondrial apoptotic pathway [20]. However, the contributions of ECs to propofol-mediated protection against I/R injury remain unknown. Here, we demonstrate that the inhibition of caveolae contributes to propofol preconditioning-suppressed EMV release and cell injury by I/R, resulting in suppression of injurious oxidative stress and apoptosis through the mitochondrial apoptotic pathway.

## 2. Methods

### 2.1. Reagents and Antibodies

Pure propofol (Sigma-Aldrich, St. Louis, MO, USA) was purchased from Sigma-Aldrich that can exclude the interference of fat emulsion. Propofol powder was dissolved into DMSO and then diluted with PBS; the final concentration of DMSO was less than 0.1% to reduce vehicle effects on cell function and experimental measures. Reagents used in the experiment were as follows: Dulbecco's modified Eagle's medium (DMEM), fetal bovine serum (FBS), penicillin, streptomycin, trypsin-EDTA (all from GIBCO Laboratories, Grand Island, New York, USA) and dimethylsulfoxide (DMSO). Antibodies used in the experiment were as follows: anti-beta-actin (sc-47,778; Santa Cruz, USA), anti-caspase 3 antibody (25546-1-AP, Proteintech, China), anti-Bax antibody (50599-2-Ig, Proteintech, China), anti-BCL2 antibody (12789-1-AP, Proteintech, China), anti-cytochrome-c antibody (66264–1-Ig, Proteintech, China), anti-COXIV antibody (11242–1-AP, Proteintech, China), anti-Caveolin-1 antibody (C4490; Sigma-Aldrich, USA), and anti-CD144/VE cadherin (62,340; LSBio, USA). Assay kits used in the experiment were as follows: Methyl-*β*-cyclodextrin (*β*-CD) (C4555, Sigma-Aldrich, USA), cell counting kit-8 (CCK-8) (CK04, Shanghai Tongren, China), mitochondrial viability stain (ab129732, Abcam, UK), lactate dehydrogenase (LDH) assay kit (A020-2, Nanjing Jiancheng Bioengineering Institute, Nanjing, China), reactive oxygen species (ROS) kit (E004, Nanjing Jiancheng Bioengineering Institute, Nanjing, China), intracellular malondialdehyde (MDA) kit (A003-4, Nanjing Jiancheng Bioengineering Institute, China), total superoxide dismutase (T-SOD) kit (A001-01, Nanjing Jiancheng Bioengineering Institute, China), JC-1 mitochondrial membrane potential detection kit (BB-4105-3, BestBio, China), cell mitochondria isolation kit (C3601, Beyotime, China), and DeadEnd™ Fluorometric TUNEL System (G3250, Promega, USA).

### 2.2. Cell Culture and Construction of the H/R Model

Human umbilical vein endothelial cells (HUVECs) were cultured in DMEM supplemented with 10% FBS, 100 units/mL penicillin, and 100 mg/mL streptomycin at 37°C under a humidified 5% CO_2_ and 95% O_2_ atmosphere.

HUVECs at 95% confluence in 10 × 10 cm^2^ vessels were exposed to H/R with or without propofol preconditioning. Cultured ECs were divided into five treatment groups: normal control (NC), hypoxia-reoxygenation (H/R), low-dose (50 *μ*mol/L) propofol preconditioning (P50) then H/R, high-dose (100 *μ*mol/L) propofol preconditioning (P100) then H/R, and DMSO (100 *μ*mol/L) vehicle control (D100).The hypoxic environment was a moist closed plastic vessel aerated with a 94% N_2_, 5% CO_2_, and 1% O_2_ mixture for 5 min before being sealed. Cells were subjected to hypoxia for 12 hours (h) at 37°C [21]. Cultures were divided into five groups: an untreated control group (NC); an H/R untreated group; and three pretreatment groups, low dose of propofol(50 *μ*mol/L) (P50 group), high-dose propofol (100 *μ*mol/L) (P100 group), and equal volume DMSO (100 *μ*mol/L) (D100group) for 4 h followed by H/R. Cells in the H/R, P50, P100, and D100 groups were cultured in glucose- and serum-free medium during H/R. In the NC group, the DMEM plus FBS was replaced by new DMEM plus FBS as a control for the media changes in the other treatment groups.

### 2.3. Cell Viability Measured by CCK-8 and LDH Assays

The agent in cell counting kit (CCK) can be restored by dehydrogenase in mitochondria and then produced highly water-soluble orange formazan product in the case of electronic coupling agent is present [21]. OD value was measured using a microplate reader at a wavelength in the 450 mM which indirectly reflected the viability of cells.

Lactate dehydrogenase (LDH) is an aglycolytic enzyme involved in pyruvate to lactic acid, which is present in almost all tissues or cytoplasm in the body. When the cell membrane was damaged, LDH was rapidly released into the cell culture medium, so we determine the degree of cell damage by detecting LDH activity in cell culture supernatant. The operating method according to the instructions and then the OD value were measured using a microplate reader at a wavelength in the 450 nm which indirectly reflected the degree of cell damage.

### 2.4. Detection of Intracellular ROS, MDA, and T-SOD

Intracellular ROS was measured by 2,7-dichlorodihydrofluorescein diacetate (DCF-DA) staining and quantified by flow cytometer. DCFH-DA is the most widely used and most sensitive intracellular ROS detection probe. DCFH-DA has no fluorescence and is hydrolyzed into DCFH (dichlorodihydrofluorescein) by esterase after the entering cells. In the presence of ROS, DCFH is oxidized into the enhanced green fluorescent substance DCF which cannot penetrate the cell membrane, and its fluorescence intensity is directly proportional to the level of intracellular ROS. The DCFH-DA was added in the medium at working concentration and incubated for 40 min under 37°C, followed by cell digestion for 3 min by trypsin; the medium with 10% FBS was added to terminate the cell digestion, and then the cell suspension was prepared 1000*g* cell suspension was centrifuged for 5 min to collect cells, which were washed for 1-2 times with PBS. It was used for FACS detection after cell sediments were suspended in PBS. The optimal excitation wavelength of fluorescence is 500 and 485 nm, and the optimal emission wavelength is 525 nm.

The level of SOD activity indirectly reflects the ability of an organism to scavenge ROS, while the level of MDA indirectly reflects the severity of cells attacked by ROS. The MDA is the degradation product of lipid peroxide which can be condensed with thiobarbituric acid (TBA), resulting in a red product which has the maximum absorption peak at 532 nm; intracellular superoxide anions can oxidize hydroxylamine, resulting in nitrite which shows purplish red under the action of a chromogenic agent. The samples in all groups were added to 96-well plates and measured with commercial assay kits for SOD and MDA, as described, respectively [22].

### 2.5. Cell Mitochondrial Isolation

After digestion with trypsin, HUVECs were collected by centrifugation and gently resuspended the cells with ice-cold PBS, then take a few cells for counting and the remaining cells were centrifugated for 600*g* at 4°C for 5 min; the supernatant was discarded and 1-2.5 ml cell mitochondrial isolated reagent was used to gently resuspended the cells, then placed it 10–15 min in ice; and then after homogenization and repeated centrifugation, the precipitate is isolated mitochondria after be careful removal of the supernatant. Once the mitochondria were separated from the cells, we can obtain the removal of mitochondrial cytoplasmic protein for the release of cytochrome c into the cytoplasm.

### 2.6. Mitochondrial Activity and Measurement of Intracellular ATP

Mitochondrial Viability Stain (ab129732) is a fluorometric/colorimetric assay that uses an indicator dye to measure oxidation-reduction reactions which principally occur in the mitochondria of living cells. The dye can be measured by a microplate reader at a wavelength in the 570 nm.

Adenosine 5′-triphosphate (ATP) is the basic carrier of energy conversion in vivo and is the most important energy molecules in the cell involved in various physiological and pathological processes. Normally, ATP levels will fall when cells befall apoptosis, necrosis, or other toxic state, and decreased ATP levels indicate mitochondrial dysfunction or decline. During apoptosis, ATP levels often drop, occurring simultaneously with the decline of the mitochondrial membrane potential.

### 2.7. Western Blot

Western blot was used for quantitative detection of Bax, BCL2, cytochrome c, caspase 3, COXIV, and CAV-1 protein expression. Protein samples were, respectively, added to 10% SDS polyacrylamide gel for electrophoresis, and then transferred to a PVDF membrane. Thereafter, the PVDF membrane was incubated together with an antibody at 4°C overnight. On the next day, the PVDF membrane was placed on a shaker for rewarming 30 min at room temperature, then washed with PBS-T for 10 min 4 times; then the PVDF membrane was incubated with corresponding secondary antibodies in the shaker at room temperature for 4 hours and washed with PBS-T for 10 min 4 times before exposure and was analyzed as described [21].

### 2.8. Assessment of Mitochondrial Membrane Potential with JC-1 Staining and Mitochondrial Membrane Permeability with MPTP

The decrease of mitochondrial membrane potential marks the early apoptosis. In the case of higher mitochondrial membrane potential, JC-1 aggregates in the mitochondrial matrix, forming a polymer with its maximum emission wavelength at 590 nm when excited at 488 nm, and red fluorescence can be emitted. In the case of lower mitochondrial membrane potential, JC-1 cannot aggregate in the mitochondrial matrix and acts as a monomer with its maximum emission wavelength at 527 nm when excited at 488 nm, and green fluorescence can be emitted. In this way, changes in the mitochondrial membrane potential can be detected based on the changes of fluorescent color. Therefore, the application of JC-1 dye has been widely used for detecting mitochondrial depolarization occurring in apoptosis. Sample cells were collected and washed twice with PBS; then the cells were collected through centrifugation, resuspended in 500 *μ*L JC-1 staining solution, and incubated for 15 min in an incubator with 5%CO_2_ under 37°C. Thereafter, the cells were collected again through centrifugation and resuspended in 500 *μ*L preheated incubation buffer. The results were detected and analyzed with flow cytometer as we described [23].

Mitochondrial permeability transition pores (MPTP) are nonspecific calcium-dependent channel composed of an inner and outer mitochondrial membrane component. MPTP will open and the permeability of the mitochondrial membrane will be significantly altered when cells undergo the state of apoptosis, necrosis, oxidative stress, and other stimulations, which will lead to the release of cytochrome c and other mitochondrial contents and cause significant fall of the mitochondrial membrane potential. Calcein was used to detect the state of MPTP through the change in fluorescence of the mitochondria.

### 2.9. Terminal Deoxynucleotidyl Transferase dUTP Nick End Labeling (TUNEL) Assay for Cell Apoptosis

Apoptotic cells were detected by TUNEL staining with a commercially available kit (G3250, Promega, USA). Apoptosis of HUVECs was quantitated by counting the number of TUNEL-positive cells in random microscopic high-power fields.

### 2.10. Collection and Preparation of EMV Samples and Analysis of EMV Morphology and Concentration

After H/R or control incubation, the membrane vesicles at the bottom of the cell culture dish were blown gently with a pipette before removal of the cell culture media. Harvested media samples were transferred into centrifuge tubes and centrifuged at 400*g* for 15 minutes at 4°C, then the supernatant was transferred into new centrifuge tubes and centrifuged at 2000*g* for 15 min at 4°C. Precipitates were discarded and the supernatant was transferred to ultra-high speed centrifuge bottles and centrifuged at 20000*g* for 70 min at 4°C [24]. After centrifugation, the culture medium was discarded, and the pelleted membrane vesicles were resuspended in 1 ml PBS. Suspended EMVs were immediately analyzed or stored at −80°C [25].

The morphologies of EMVs were assessed by transmission electron microscopy (TEM). Particle size distribution and concentration were assessed by real-time visual detection using a nanoparticles trace analyzer (NTA) according to the Brownian motion. NTA can detect vesicles with a minimum particle size of 50 nm, more sensitive than flow cytometry [26]. EMVs' surface contains many antigen epitopes; CD144 is one of the most commonly used antibodies for identification of EMVs [27]. Fluorescence microscopy can be used to identify EMVs through CD144—the membrane of EMVs' specific antigen [28].

### 2.11. Coculture Assay of Labeled EMVs with HUVECs

EMVs were labeled with PKH26 according to protocol with some modifications about centrifugal data. After the ultra-high speed centrifugation EMVs, the supernatant was discarded and resuspended with 2 *μ*M PKH26, then EMVs were labeled with PKH26 for 5 min at room temperature, and then an equal volume of 1% bovine serum albumin (BSA) was added to stop staining. After ultracentrifugation again and resuspended with culture medium, the labeled EMVs were added to HUVECs seeded in cell culture dish for 4 h incubation. The communication between EMVs and HUVECs was observed under fluorescence microscopy [29].

### 2.12. Data Analysis and Statistics

All data are presented mean ± SD. All were statistically analyzed and were conducted using SPSS 13.0 software (SPSS, Chicago, IL, USA). Group mean differences were compared by one-way ANOVA with Bonferroni correction for pairwise comparisons. Correlations between variables were assessed by Spearman's coefficient. Differences with *p* < 0.05 were considered statistically significant.

## 3. Results

### 3.1. The Decrease of Cell Viability and the Increase of Oxidative Stress Are Suppressed by Propofol Preconditioning

**Figure 1:**
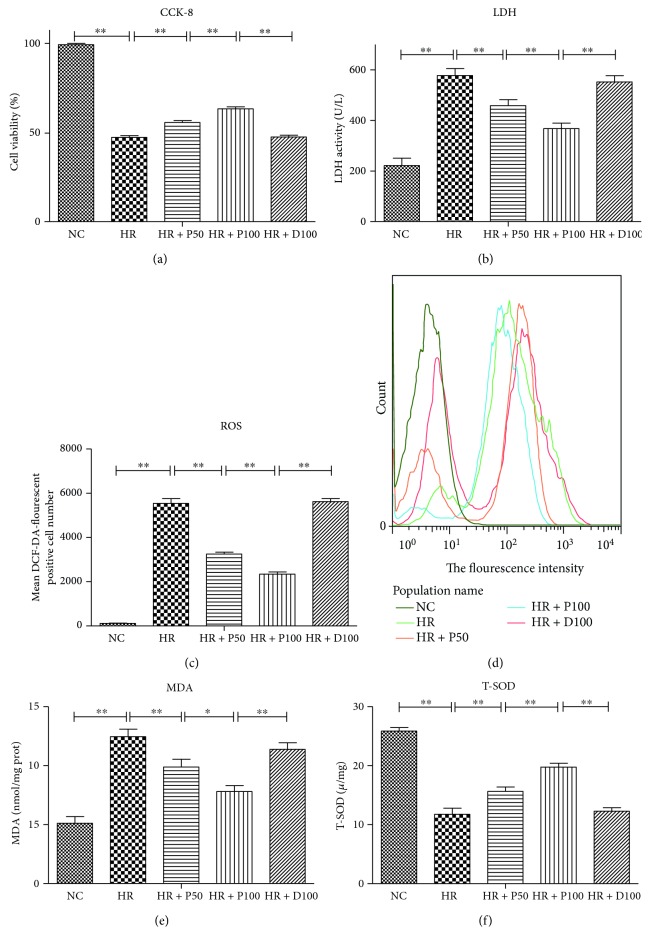
Effects of different concentrations of propofol on H/R-induced cellular damage and oxidative stress of HUVECs. (a) Different concentrations of propofol preconditioning ameliorated H/R-induced cell viability measured by CCK-8. (b) Different concentrations of propofol preconditioning ameliorated H/R-induced cell damage measured by LDH. (c, d) Different concentrations of propofol preconditioning ameliorated H/R-induced oxidative stress measured by ROS and its fluorescence intensity. (e, f) Different concentrations of propofol preconditioning ameliorated H/R-induced oxidative stress measured by MDA and T-SOD. Mean ± SD are from 5 different experiments. ∗ refers to *p* < 0.05, ∗∗ refers to *p* < 0.01.

The construction of the *in vitro* cellular H/R model was described in Methods. As shown in Figure 1, H/R significantly decreases the cell viability but increases the level of LDH (*p* < 0.01) (Figures 1(a) and (b)). The ROS production, the fluorescence intensity of DCFH, and the level of MDA indicators of oxidative stress in the cell are dramatically repressed by propofol preconditioning (*p* < 0.01) (Figures 1(c), (d), and (e)). But the total SOD (T-SOD) activity is sharply reduced to one half under this condition (Figure 1(f)) (*p* < 0.01). The H/R-induced damages are drastically attenuated by treatment with propofol dose dependently, and 100 *μ*M propofol conferred better protective effect (*p* < 0.01). As a control, the equivalent solvent DMSO does not affect the above parameters (Figure 1(f)).

### 3.2. Propofol Preconditioning Inhibits H/R-Induced Changes in Mitochondrial Apoptosis Pathway and the Expression of CAV-1

**Figure 2:**
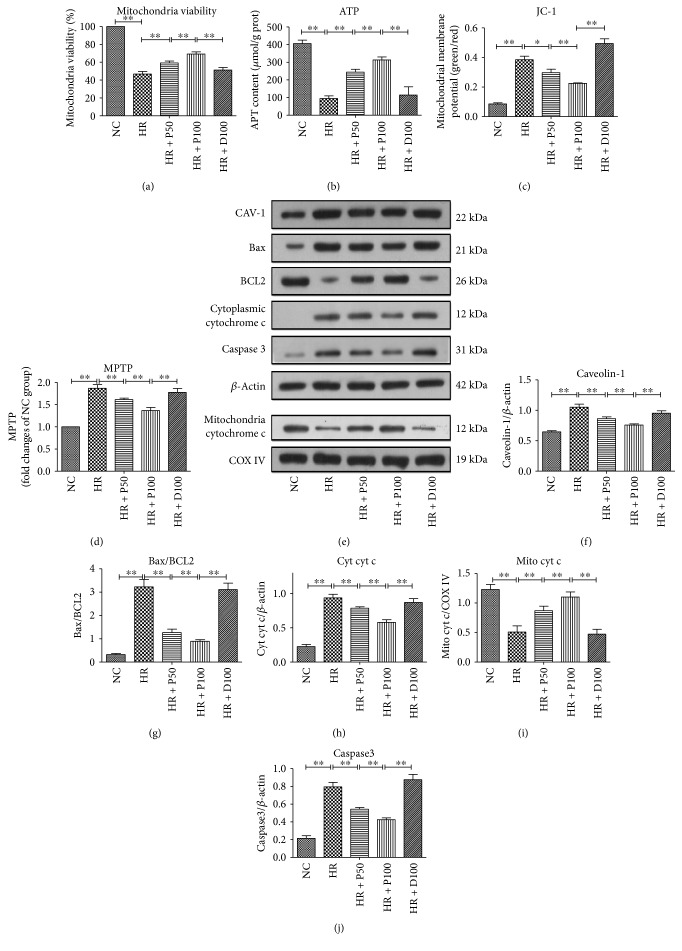
Effects of different concentrations of propofol on H/R-induced mitochondrial damage and apoptosis. (a) Different concentrations of propofol preconditioning ameliorated H/R-induced mitochondrial viability. (b) Different concentrations of propofol preconditioning enhanced the H/R-induced mitochondrial activity measured by intracellular ATP content. (c) Different concentrations of propofol preconditioning ameliorated H/R-induced decrease of mitochondrial membrane potential. (d) The effects of different concentrations of propofol preconditioning on H/R-induced mitochondrial membrane permeability. (e–j) Detecting the expression level of CAV-1, Bax, BCL2, cytochrome c, caspase 3, COX IV, and their gray scales calculated by ImageJ software. Mean ± SD are from 5 different experiments. ∗ refers to *p* < 0.05, ∗∗ refers to *p* < 0.01.

Previous evidence suggested that the mitochondrial apoptosis pathway and CAV-1 protein are involved in the damage caused by H/R-induced oxidative stress [7, 30, 31]. But how they are affected by propofol is unknown. As shown in Figure 2, H/R markedly decreases the mitochondrial viability (Figure 2(a)) (*p* < 0.01), content of intracellular ATP (Figure 2(b)) (*p* < 0.01), mitochondrial membrane potential (MMP) as measured by JC-1 dyeing (Figure 2(c)) (*p* < 0.01), the opening of MPTP (Figure 2(d)) (*p* < 0.01), and the protein levels of mitochondrial cytochrome c (mito cyt c) (Figure 2(i)) (*p* < 0.01). In addition to that, H/R also induces significant increases in the protein levels of CAV-1 (Figure 2(f)) (*p* < 0.01), ratio of Bax/BCL2 (Figure 2(g)) (*p* < 0.01), cytoplasmic cytochrome c (cyt cyt c) (Figure 2(h)) (*p* < 0.01), and caspase 3 (Figure 2(j)) (*p* < 0.01). The H/R-induced cellular and mitochondrial damages are considerably attenuated by treatment with propofol dose dependent, and 100 *μ*M propofol shows better protective effect.

### 3.3. Caveolae Inhibitor Reinforces the Protective Effects of Propofol Preconditioning against H/R-Induced Cellular Damage and Oxidative Stress

It has been shown that caveolae are involved in H/R injury in ECs. To investigate whether or not caveolae play a critical role in propofol protective effects against HUVECs H/R injury and oxidative stress, cells were initially prepared as aforementioned and treated without or with the caveolae inhibitor *β*-CD (10 *μ*mol/L) (added 1 h before propofol preconditioning) .

**Figure 3:**
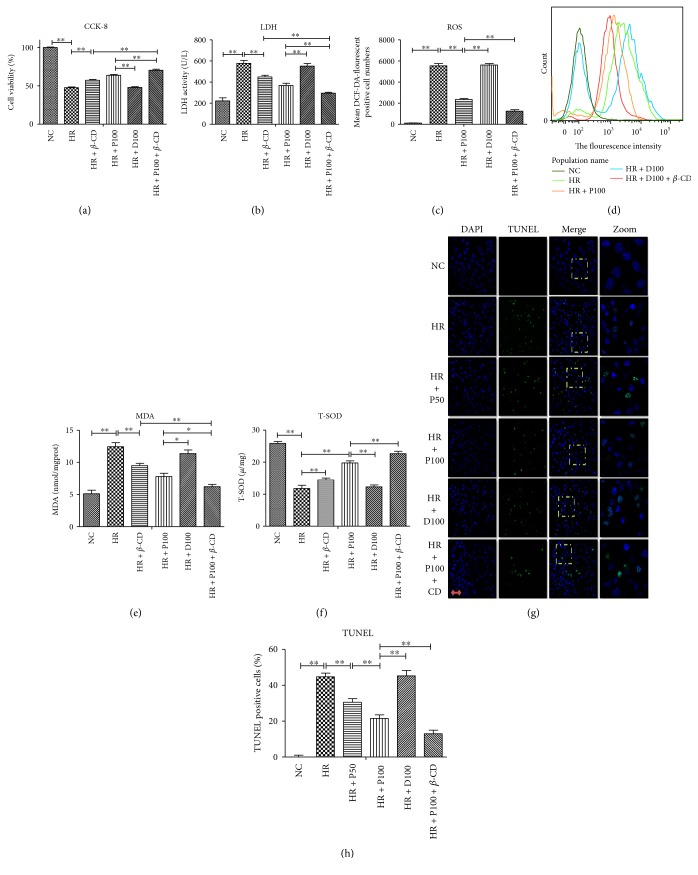
Caveolae inhibitor reinforces the protective effects of propofol preconditioning against H/R-induced cellular damage and oxidative stress. (a, b) Caveolae inhibitor reinforces the protective effects of propofol preconditioning against H/R-induced cell damage. (c, d) Caveolae inhibitor reinforces the protective effects of propofol preconditioning against H/R-induced oxidative stress measured by ROS and its fluorescence intensity. (f–h) Caveolae inhibitor reinforces the protective effects of propofol preconditioning against H/R-induced oxidative stress measured by MDA and T-SOD. (g–h) The protective effects of propofol preconditioning against H/R-induced cell apoptosis measured by TUNEL, scale bar is 5 *μ*m. Mean ± SD are from 5 different experiments. ∗ refers to *p* < 0.05, ∗∗ refers to *p* < 0.01.

As shown in Figure 3, H/R induces obvious cellular damage and oxidative stress, and all these changes are substantially attenuated by propofol treatment. Interestingly, the beneficial effects of propofol are reinforced by caveolae inhibitor *β*-CD. Unlike propofol pretreatment alone and H/R group treated with *β*-CD, propofol combined with *β*-CD pretreatment significantly increases cell viability as measured by CCK-8 and LDH activity (Figures 3(a) and 3(b)) (*p* < 0.01), concomitant with marked reductions in oxidative stress as indicated with DCF-DA, MDA, and T-SOD (Figures 3(c), 3(d), 3(e), and 3(f)) (*p* < 0.01). In addition, the beneficial effects of propofol preconditioning against H/R-induced cell apoptosis as measured by TUNEL are reinforced by caveolae inhibitor *β*-CD (Figures 3(g) and 3(h)) (*p* < 0.01).

### 3.4. Caveolae Inhibitor Enhances the Protective Effects of Propofol Preconditioning against H/R-Induced Mitochondrial-Dependent Apoptosis and Decreases the Number of EMVs in the Cell Culture Medium

The above results suggest that the protective effects of propofol preconditioning against H/R-induced injury are closely related to caveolae, but it is unknown yet whether caveolae will affect mitochondrial apoptosis pathway during H/R with propofol preconditioning.

**Figure 4:**
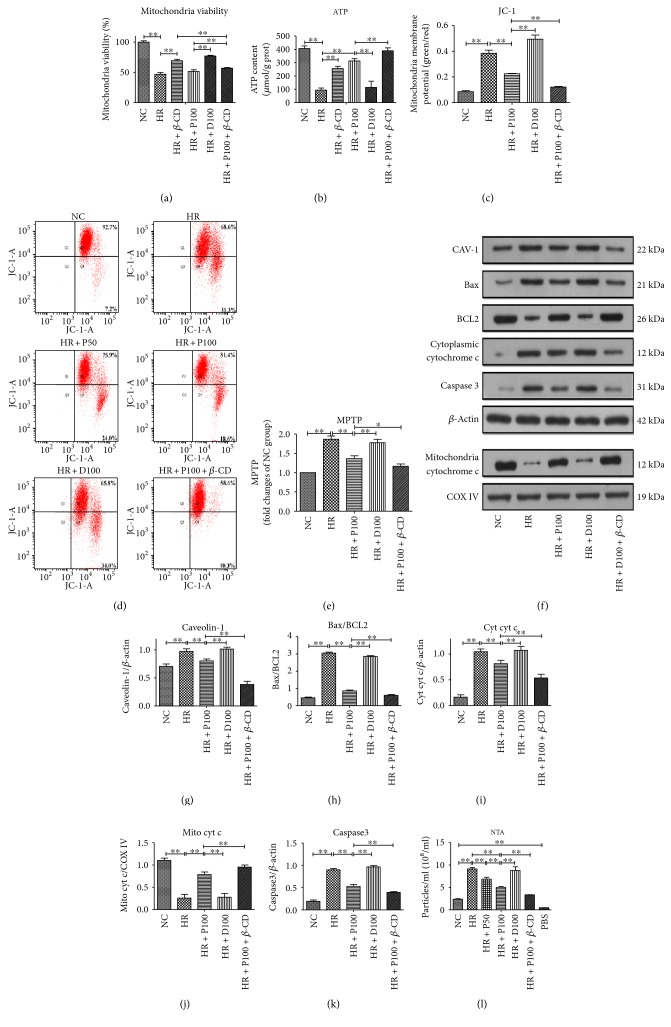
Caveolae inhibitor enhances the protective effects of propofol preconditioning against H/R-induced mitochondrial-dependent apoptosis and the release of EMVs. (a, b) The protective effects of propofol preconditioning against H/R-induced mitochondrial damage measured by mitochondrial viability and intracellular ATP content are enhanced by caveolae inhibitor. (c, d) The protective effects of propofol preconditioning against H/R-induced decrease of mitochondrial membrane potential measured by JC-1 stain and by flow cytometry. (e) The protective effects of propofol preconditioning against H/R-induced increase of mitochondrial membrane permeability are enhanced by caveolae inhibitor. (f–k) Detecting the expression level of CAV-1, Bax, BCL2, cytochrome c, caspase 3, COX IV, and their gray scales calculated by ImageJ software. (l) The protective effects of propofol preconditioning against H/R-induced release of EMVs are enhanced by caveolae inhibitor. Mean ± SD are from 5 different experiments. ∗ refers to *p* < 0.05, ∗∗ refers to *p* < 0.01.

Similar to the above data, mitochondrial viability and intracellular ATP are greatly increased by the combined use of propofol pretreatment with *β*-CD (Figures 4(a) and 4(b)) (*p* < 0.01). The beneficial effects of propofol preconditioning against H/R-induced mitochondrial-dependent apoptosis are reinforced by caveolae inhibitor *β*-CD, including mitochondrial membrane potential (MMP) as measured by JC-1 dyeing, the opening of MPTP, and the protein levels of mitochondrial cytochrome c (mito cyt c). In addition to that, combined use of *β*-CD also induces significant decreases in the protein levels of CAV-1, ratio of Bax/BCL2, cytoplasmic cytochrome c (cyt cyt c), and caspase 3 (Figures 4(a), 4(b), 4(c), 4(d), 4(e), 4(f), 4(g), 4(h), 4(i), 4(j), and 4(k)). Similar to apoptosis, the number of EMVs is greatly decreased in the presence of caveolae inhibitor *β*-CD (Figure 4(l)) (*p* < 0.01).

### 3.5. EMVs Released from H/R-Treated ECs Cause Damages to Normal HUVECs

**Figure 5:**
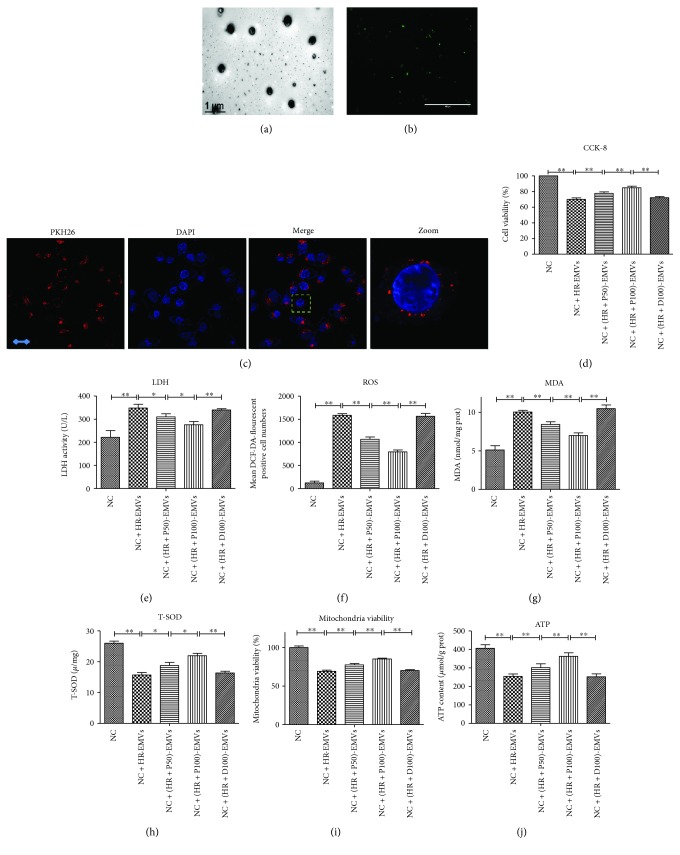
EMVs released from H/R-treated ECs cause damages to normal HUVECs. (a) Identification of EMVs by transmission electron microscopy, scale bar = 1 *μ*m. (b) Identification of EMVs by fluorescence microscopy, scale bar = 1000 *μ*m. (c) The PKH26-labeled H/R-EMVs were added to normal HUVECs for 4 h and then observed by confocal. Scale bar = 2 *μ*m. (d, e) H/R-EMVs caused cell damage to normal HUVECs measured by CCK-8 and LDH. (f–h) H/R-EMVs caused oxidative stress to normal HUVECs measured by ROS, MDA, and T-SOD. (i–j) H/R-EMVs caused mitochondrial damage to normal HUVECs measured by mitochondrial viability and intracellular ATP content. Mean ± SD are from 5 different experiments. ∗ refers to *p* < 0.05, ∗∗ refers to *p* < 0.01.

With the restoration of blood flow of reperfusion, EMVs released by ECs in the ischemic area would flow to normal vascular ECs, but it is unclear if normal ECs can be influenced by EMVs released from H/R-treated cells. The EMVs were first isolated and characterized by electron microscopy and fluorescence labeled (Figures 5(a) and 5(b)). Then, the PKH26-labeled EMVs were added to normal HUVECs for 4 h and the PKH26 fluorescence can be observed in HUVECs, which manifests that EMVs could be transmitted to HUVECs and may pass information to HUVECs (Figure 5(c)) [11, 12, 32]. Indeed, EMVs released from H/R-treated cells reduce the cell viability of targeted HUVECs (Figure 5(d)) (*p* < 0.01), the level of T-SOD (Figure 5(h)) (*p* < 0.01), mitochondrial activity (Figure 5(i))(*p* < 0.01), and intracellular ATP (Figure 5(j)) (*p* < 0.01), concomitant with significant upregulation in level of LDH (Figure 5(e)), ROS (Figure 5(f)) (*p* < 0.01), and MDA (Figure 5(g)) (*p* < 0.01).

## 4. Discussion

Here, we have shown that propofol preconditioning not only ameliorates the cellular and mitochondrial damages in the endothelium cells but also inhibits the release of microvesicles by destroying the structure of the caveolae during hypoxia-reoxygenation condition. During either H/R or propofol preconditioning, the expression of CAV-1 is corelated with the other apoptotic proteins. We also found that the microvesicles induced by hypoxia-reoxygenation could transmit between cells, which might increase the cellular level of ROS and cause dysfunction of mitochondria in target cells.

Previous reports have shown that the increased expression of CAV-1 leads to the decreased activity of antioxidative enzymes and finally induces cell apoptosis [31, 33–35]. On the contrary, inhibition of CAV-1 enhances the enzymatic activity of those proteins and ameliorates the cellular damage under ischemia/reperfusion condition. Although propofol is a widely used clinical drug with antioxidative activity in ischemia/reperfusion, its mechanism of action remains unknown [36, 37]. As we know that hypoxia-reoxygenation stimulates the production of microvesicles in EMVs and that EMVs play an important role in intercellular communication and information exchange during a variety of physiological and pathological process, such as procoagulant and proinflammatory signals [38], but how they are affected by caveolae is not clear [39, 40]. Our study for the first time investigated the connection between caveolae, microvesicles, and hypoxia-reoxygenation. We demonstrated that propofol suppresses cellular apoptosis and downregulates the level of CAV-1, the marker protein of caveolae, and the latter when inhibited, decreases the number of EMVs in the cell culture medium. The reason why inhibition of caveolae reduces the number of EMVs is not clear. Caveolin-1 is a membrane-integrated protein with a unique structure, and it can help to form membrane curvature during membrane invagination [41]. Extracellular vesicles including microvesicles are budding from the plasma membrane, and they need the help of some membrane-shaping proteins, like caveolin-1, to form the curvature. Indeed, some researchers found that caveolin-1 is present in isolated extracellular vesicles from cancer cells, suggesting that caveolin-1 may contribute to the formation of extracellular vesicles [42]. This is partly explained in our study that the caveolae inhibitor *β*-CD is able to reduce the number of microvesicles.

Compared to the observations from others, we provide new evidence showing that the microvesicles from HR-treated ECs may be able to transmit some adverse messages to target cells, which causes the increase of ROS and dysfunction of mitochondria in ECs.

However, some contradictory results also indicate CAV-1 is downregulated in hepatic I/R, where it can contribute to I/R injury [17, 43]. The discrepancy may be due to the different cellular models and various expression patterns of CAV-1. How is microvesicle formed and what is the content inside becomes a very hot topic attracting massive interest from scientist worldwide during the recent years. However, there are still a lot of unknowns. For example, is the beneficial effect of propofol preconditioning (antioxidative stress) in ECs is either due to the decreased release or the changed contents of the microvesicle under H/R conditions? So far, no one can exactly answer this question. Therefore, more studies need to be done in the future. We believe that EMVs are promising markers for diagnosis, evaluation of disease development, treatment, and clinical prognosis, which may be used to evaluate the changes in endothelial cell function.
